# Takotsubo Cardiomyopathy in the context of psychiatric illness: a review of case-based and research evidence

**DOI:** 10.1192/j.eurpsy.2026.12021

**Published:** 2026-07-31

**Authors:** A.-L. Comșa, I. C. Mândraș

**Affiliations:** 1 Psychology and Educational Sciences, Babes-Bolyai University; 2 Psychiatry, County Clinical Energency Hospital, Cluj-Napoca , Romania

## Abstract

**Introduction:**

Takotsubo cardiomyopathy (TC), or stress cardiomyopathy, is a transient left ventricular dysfunction resembling acute coronary syndrome without obstructive coronary disease. Emotional or physical stress often triggers TC, and pre-existing psychiatric disorders appear to increase susceptibility compared to healthy controls or patients with other heart conditions.

**Objectives:**

This review aims to synthesize current evidence from case reports and research studies on the presentations, clinical features, and implications of TC in psychiatric patients, highlighting diagnostic challenges and avenues for improved management.

**Methods:**

We conducted a literature search in PubMed and ScienceDirect using a previously established search string. Eligible studies were required to be original research articles published in English between 2016 and 2025, and to address the association between TC and psychiatric conditions. In total, 41 articles met the inclusion criteria.

**Results:**

Mood disorders, particularly major depressive disorder and bipolar disorder, have emerged as relevant comorbidities in patients who develop TC. Clinical observations indicate that depressive episodes, whether unipolar or within the bipolar spectrum, can act as both a chronic vulnerability factor and an acute trigger context for cardiac dysfunction. Furthermore, TC has been associated with additional psychiatric conditions, including psychotic disorders, personality disorders, and substance use disorders. Reports have associated serotonin-norepinephrine reuptake inhibitors with catecholamine surges, while lithium has likewise been implicated via its sympathomimetic influence on catecholamine release. Even electroconvulsive therapy has occasionally triggered TC events despite otherwise stable cardiovascular profiles. In psychiatric contexts, the clinical presentation of TC can be particularly challenging to identify, as symptom manifestations often overlap with psychiatric or medication-related phenomena. Patients may present with classic cardiologic complaints, but these can be often underreported.

**Conclusions:**

Bringing mental health expertise into cardiovascular care pathways for patients vulnerable to TC requires structured collaboration that extends across acute, subacute, and recovery phases. The evidence consistently suggests that psychiatric comorbidity is not merely incidental but an active modifier of both disease onset and recurrence risk. Evaluating the safety and impact of psychotropic agents in this context, as well as refining procedural protocols to minimize iatrogenic triggers, will enhance clinical management. Ultimately, advancing care for individuals affected by TC requires a holistic approach that integrates cardiovascular and mental health perspectives, recognizing the dynamic interplay between emotional stress, neuroendocrine function, and myocardial vulnerability.

**Disclosure of Interest:**

None Declared

